# Enhanced Transcriptional Signature and Expression of Histone-Modifying Enzymes in Salivary Gland Tumors

**DOI:** 10.3390/cells12202437

**Published:** 2023-10-11

**Authors:** Maria Manou, Theodoros Loupis, Dimitrios M. Vrachnos, Nikolaos Katsoulas, Stamatios Theocharis, Dimitrios S. Kanakoglou, Efthimia K. Basdra, Christina Piperi, Athanasios G. Papavassiliou

**Affiliations:** 1 Department of Biological Chemistry, Medical School, National and Kapodistrian University of Athens, 11527 Athens, Greece; maria_manou@hotmail.com (M.M.); kanakogloud@biol.uoa.gr (D.S.K.); ebasdra@med.uoa.gr (E.K.B.); 2 Haematology Research Laboratory, Clinical, Experimental Surgery and Translational Research Center, Biomedical Research Foundation, Academy of Athens, 11527 Athens, Greece; tloupis@bioacademy.gr (T.L.); dimvrachnos@gmail.com (D.M.V.); 3 First Department of Pathology, Medical School, National and Kapodistrian University of Athens, 11527 Athens, Greece; nikolaoskatsoulas@gmail.com (N.K.); stamtheo@med.uoa.gr (S.T.)

**Keywords:** salivary gland tumors, histone modifications, SUV39H1, EZH2, HDAC8, p53

## Abstract

Salivary gland tumors (SGTs) are rare and complex neoplasms characterized by heterogenous histology and clinical behavior as well as resistance to systemic therapy. Tumor etiology is currently under elucidation and an interplay of genetic and epigenetic changes has been proposed to contribute to tumor development. In this work, we investigated epigenetic regulators and histone-modifying factors that may alter gene expression and participate in the pathogenesis of SGT neoplasms. We performed a detailed bioinformatic analysis on a publicly available RNA-seq dataset of 94 ACC tissues supplemented with clinical data and respective controls and generated a protein–protein interaction (PPI) network of chromatin and histone modification factors. A significant upregulation of *TP53* and histone-modifying enzymes SUV39H1, EZH2, PRMT1, HDAC8, and KDM5B, along with the upregulation of DNA methyltransferase DNMT3A and ubiquitin ligase UHRF1 mRNA levels, as well as a downregulation of lysine acetyltransferase KAT2B levels, were detected in ACC tissues. The protein expression of p53, SUV39H1, EZH2, and HDAC8 was further validated in SGT tissues along with their functional deposition of the repressive histone marks H3K9me3 and H3K27me3, respectively. Overall, this study is the first to detect a network of interacting proteins affecting chromatin structure and histone modifications in salivary gland tumor cells, further providing mechanistic insights in the molecular profile of SGTs that confer to altered gene expression programs.

## 1. Introduction

Malignancies of salivary gland (SG) origin are rare neoplasms, constituting 0.5–1.2% of total cancers, and approximately 3–6% of all head and neck (HN) tumors [1,2]. They are characterized by histological diversity and clinical behavior as well as resistance to therapy and often poor prognosis of highly malignant cases [3].

Although the specific etiologic factors that contribute to the development of salivary gland tumors (SGTs) are still under elucidation, there is evidence that their heterogeneity is attributed to a range of genetic alterations in addition to histopathological and morphological characteristics [4,5]. Chromosomal deletions in the 9p21 (*CDKN2A* gene) and 12q12-q13 (*NAB2*, *ERBB3* genes encoding for keratins/type I and II) regions present a high incidence on subtypes of biphasic (epithelial/myoepithelial) nature, such as adenoid cystic carcinomas (ACCs) [6], and along with alterations in *MYB* gene expression may confer to the proliferative, differentiative, and apoptotic properties of tumor cells [7]. In addition, a high expression of p53 is often observed in mucoepidermoid carcinomas (MECs) and higher-grade ACCs [8,9,10,11,12], while p53 modifications have been associated with the transformation process to a higher grade, often due to mutations of *TP53*, or heterozygosity loss on gene loci [8,9]. Additional mutations affecting NOTCH pathway genes have been detected in cases of fusion-negative ACCs [6], while upregulation of ID1 and its target genes (*CDKN2A*, *MMP1*, *S100A9*) is involved in the metastatic potential of SGTs [13].

An interplay of genetic and epigenetic changes has been recently suggested to contribute to SGT pathogenesis, characterized by aberrant DNA methylation and deregulated expression of histone-modifying enzymes [14,15,16,17,18]. DNA methylation of cyclin-dependent kinase inhibitors (CKI) and *p27*, *p21*, *p19*, *p18*, and *p15* genes is frequently observed in ACCs, being considered as an early event in tumor development [19], while the methylation of *RARb2* and *RASSF1* and promoter hypermethylation of *p16(INK4A)* have been associated with ACC pathogenesis [5]. Experimental in vitro and in vivo data have further revealed that methylation of the cadherin-1 (*CDH1)* gene, which codes for epithelial cadherin (E-cadherin), correlates with the differentiative and invasive properties observed in SGTs [20,21].

Regarding histone modifications, whole-exome sequencing has revealed that 35–50% of ACCs harbor variations related to chromatin-remodeling genes (*CHD2*, *KDM5A*, *SMARCA2*, *BRD2*) [22,23]. Additionally, elevated expression of several methylation and acetylation histone marks H3K9ac/K9me3 and H3K18ac has been detected in salivary gland neoplasms which were further correlated with the aggressive characteristics of high-grade tumors [24]. In concert, a recent study from our group investigating the expression of HDAC-1, -2, -4, and -6 in malignant SGTs and potential correlations with clinicopathological characteristics detected increased immunohistochemical expression of HDAC-1 and HDAC-2 in low- and high-grade ACCs, respectively. Of interest, HDAC-2 expression was positively correlated with favorable prognosis while HDAC-6 upregulation was associated with worse patient prognosis, suggesting a potential therapeutic target [18].

This study aims to detect, in a more systematic way, epigenetic regulators and histone-modifying factors that may alter gene expression and participate in the pathogenesis of SGT neoplasms. To this end, we have performed detailed bioinformatic analysis on a publicly available RNA-seq dataset of ACC tissues supplemented with clinical data and respective controls [25]. We have generated a protein–protein interaction (PPI) network of key histone modification factors and validated the expression of selective histone-modifying enzymes along with their respective histone marks in SGT tissue samples.

## 2. Materials and Methods

### 2.1. Public Data Acquisition

To investigate the key epigenetic regulators and histone modifiers that might contribute to the pathology of SGTs, we obtained publicly available RNA-Seq data (Bioproject accession number PRJNA287156) [25] from the National Institute of Health’s (NIH) Sequence Read Archive (SRA) [26,27]. The dataset consists of 98 samples from archived FFPE tissue slides, of which 94 refer to salivary adenoid cystic carcinoma (ACC) tissue and 4 to normal salivary glands, sequenced on Ion Proton and Ion S5/XL systems.

### 2.2. Analyzing RNA Sequencing Data

Processing of sequencing reads of RNA-Seq data was performed using in-house computational pipelines. The read quality of the raw single-end FASTQ files was evaluated using FastQC (*version 0.11.7*) [28]. Adapter, quality, and polyX trimming was carried out using fastp (*version 0.20.0*) [29]. Reads were mapped on a decoy-aware transcriptome (gencode41/GRh38) and gene expression count was performed using Salmon (*version 1.1.0*). To uphold robustness in our analysis, we dismissed any samples that recorded fewer than 2 million mapped reads from the subsequent stages of the study.

### 2.3. Differential Gene Expression Analysis and Functional Enrichment Analysis

The data were normalized using the variance-stabilizing transformation function offered by the DESeq2 package (*version 1.36.0*) [30] in R (*version 4.2.1*). The aforementioned R package was also used to identify differentially expressed genes (DEGs) with these specific criteria: Log_2_FC > 1 or <−1, and adjusted *p*-value < 0.05. The DEGs were further used for gene set enrichment analysis (GSEA) and over-representation analysis (ORA) using the WEB-based Gene Set Analysis Toolkit (WebGestalt, http://www.webgestalt.org, accessed on 8 November 2022) [31] in order to elucidate their biological context. ORA and GSEA offer a statistical evaluation of the proportion of genes within a specified pathway that appears in an altered expression gene set. Our enriched terms of interest were gene ontology (GO) terms related to biological processes, cellular components, and molecular functions [32], as well as KEGG biological pathways [33]. The statistical significance of the over-representation of biological terms was calculated using a hypergeometric distribution, with *p*-values adjusted using the false discovery rate (FDR) method. Terms with adjusted *p*-values < 0.05 were deemed statistically significant.

Network analysis of the DEGs was performed using Cytoscape (*version 3.9.1*) [34] and the stringApp plugin (*version 2.0.1*) [35], a Cytoscape plugin that allows import of STRING networks [36] of PPIs. Finally, to ensure our focus on genes involved in histone modifications, we intersected the DEG network with a second network, generated by querying *‘histone modification*’ in the stringApp. This intersection resulted in a network consisting of DEGs implicated in histone modifications and are known to interact with each other.

Lastly, we employed pathview (*version 1.40.0*), a tool designed for pathway-based data integration and visualization, to map and render diverse biological data onto relevant pathway graphs [37].

### 2.4. Patients’ Tissues Description

Medical records and archival histopathological material of 10 SGT patients were used to validate the computational transcriptome data. All patients had been diagnosed in the First Department of Pathology, Laikon Hospital, National and Kapodistrian University of Athens between 2013 and 2021. In all cases, the diagnoses and grading were peer-reviewed according to the principles laid down in the World Health Organization (WHO) classification of SGTs. Written informed consent from all patients was available and the study has been approved by the Bioethics and Ethics Committee of the National and Kapodistrian University of Athens (approval number: 108). The study sample consisted of 2 benign tumors (pleiomorphic adenomas, PA) and 8 malignant tumors (5 adenoid cystic carcinomas, ACC, and 3 polymorphous adenocarcinomas, PAC). Four of the patients (40%) were male and six (60%) were female. Overall mean age at diagnosis was 52.1 years for benign and 70.5 years for malignant SGT patients, respectively.

### 2.5. Immunohistochemistry

Formalin-fixed, paraffin-embedded (FFPE) SGT tissue sections (4 μm) were dewaxed in xylene and brought to water after degraded alcohol solutions. Antigen retrieval was performed by microwaving tissue slides in suitable buffer for 15 min at high power. To remove the endogenous peroxidase activity, sections were then treated with freshly prepared hydrogen peroxide (dark for 10 min/room temperature). Nonspecific antibody binding was blocked using 5% normal goat serum (NGS) for 1 h. Tissue sections were incubated with primary antibody overnight (4 °C) against Ki-67, clone MIB-1, Ph9, 1:200 (Dako, Santa Clara, CA, USA); Anti-trimethyl-Histone H3(Lys27), rabbit polyclonal, pH6, 1:50 (Millipore, Darmstadt, Germany); Anti-trimethyl-Histone H3(Lys9) clone 6F12-H4 mouse monoclonal IgG1, pH6, 1:80 (BioLegend, London, UK); SUV39H1, clone MG44, Ph6, 1:300 (Millipore); EZH2, clone 11-EZH2, pH9, 1:100 (Abcam, Cambridge, UK); HDAC8, pH6, 1:150 (Abcam). Sections were then incubated at room temperature with biotinylated linking reagent, followed by incubation with peroxidase-conjugated streptavidin label. As a visualization system, we used EnVision FLEX, Dako, according to the manufacturer’s instructions. The resultant immune peroxidase activity was developed using diaminobenzidine (DAB) substrate kit according to the manufacturer’s instructions. Sections were counterstained with Harris’ hematoxylin and suitable positive controls were implemented as well. Table 1 summarizes the characteristics of each antibody used for immunohistochemistry.

### 2.6. Evaluation of Immunohistochemistry

Evaluation of immunohistochemical reactivity was performed by two independent pathologists (S.T; Ν.Κ) with no knowledge of clinical data. Samples were considered as positive (when >5% of tumor cells within the sample were stained), while the intensity of the immunoreaction was evaluated by further subcategorization as low, moderate, or intense (0: negative staining, 0–4%, 1: 5–24%, 2: 25–49%, and 3: 50–100%) and staining intensity was scored as 0: negative; 1: mild; 2: intermediate; or 3: intense staining. A labeling index based on the percentage of stained neoplastic cells was calculated.

## 3. Results

### 3.1. Increased Transcriptional Signature of Chromatin-modifying Enzymes in ACC Tumors

Employing bioinformatics analysis, we identified key differentially expressed genes (DEGs) and delineated their significant interactions, specifically those involved in histone modifications. Initially, we obtained the list of DEGs from the DESeq2 package (Supplementary Table S1). Functional enrichment analysis of the DEG lists produced lists of statistically significant GO biological processes, cellular components, and molecular processes, as well as KEGG biological pathways.

**Figure 1:**
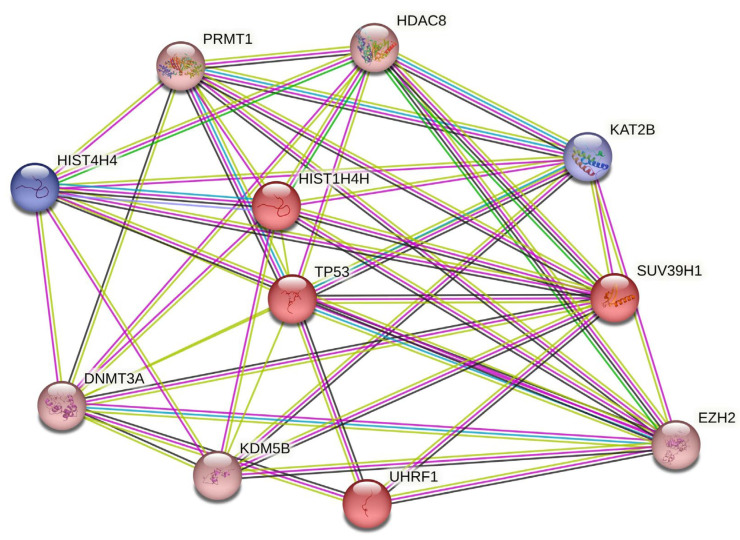
Intersected PPI network depicting DEGs involved in histone modifications. This network is the product of intersecting the DEG network with a second one, generated from querying ‘*histone modification*’ in the stringApp. The nodes of the network, each representing all the proteins produced by a single protein-coding gene locus, symbolize proteins. Splice isoforms or post-translational modifications are collapsed in order to streamline interpretation. Nodes colored in blue are downregulated and nodes colored in red are upregulated, and they both indicate proteins with known or predicted 3D structures. Each connection, or edge, in this network symbolizes a relationship between two proteins, categorized as follows: (a) known interactions, represented in cyan for curated databases and purple for experimentally determined; (b) predicted interactions, marked in light green for gene neighborhood, red for gene fusions, and blue for gene co-occurrence; and (c) other interactions, highlighted in yellow for text mining, black for co-expression, and light blue for protein homology. It is important to note that these associations do not necessarily indicate direct physical binding between the proteins involved.

Subsequently, network generation was carried out using the Cytoscape tool, involving an intersection of the DEG network with another network, created by querying *‘histone modification*’ in the stringApp. This process resulted in an enriched PPI network (Figure 1), highlighting crucial genes implicated in histone modifications and ACC pathology. This network, as reported by STRING, consists of 11 nodes and 46 edges, with an average node degree of 8.36 and an average local clustering coefficient of 0.875. Furthermore, the expected number of edges was only seven, which underscores the significance of the PPI enrichment *p*-value of <1 × 10^−16^, indicating that the network’s observed interactions are not random and that the proteins are at least partially biologically connected as a group.

The PPI network indicates a significant upregulation of the *TP53* gene in ACC neoplasms interacting with several histone-modifying enzymes, such as the lysine methyltransferases SUV39H1 and EZH2, the protein arginine methyltransferase 1 PRMT1, the lysine demethylase KDM5B, and the histone deacetylase HDAC8. An upregulation was also detected in DNA Methyltransferase 3 Alpha (DNMT3A) and ubiquitin ligase UHRF1, while a significant downregulation was revealed in lysine acetyltransferase KAT2B expression levels. Table 2 summarizes the detected genes involved in the PPI network followed by their expression levels and respective statistical significance. The enriched terms of the resulting PPI network can be found in Supplementary Table S2. The STRING analysis can be reached via the permanent link: https://version-11-5.string-db.org/cgi/network?networkId=bJlnNgyoEmk4 (accessed on 7 October 2023).

### 3.2. p53 and Histone-Modifying Enzyme Protein Expression Is Upregulated in SGT Tissues

**Figure 2:**
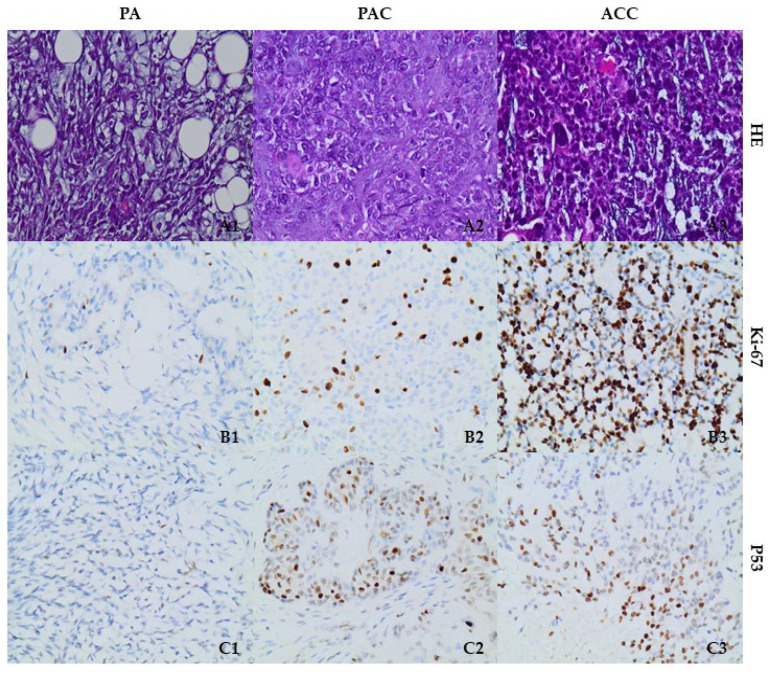
Representative immunostainings of H&E, Ki-67, and p53 in a PA case (**A1**,**B1**,**C1**), in a PAC case (**A2**,**B2**,**C2**), and in an ACC case (**A3**,**B3**,**C3**). Magnification ×400.

Following the transcriptional data analysis, we proceeded to evaluate the protein expression levels of p53 and histone-modifying enzymes SUV39H1, EZH2, and HDAC8 in 10 tissues of salivary gland tumors, including 2 pleiomorphic adenomas, PA, 3 polymorphous adenocarcinomas, PAC, and 5 adenoid cystic carcinomas, ACC. The cell proliferation activity was examined using Ki-67 staining in all SGT tissues. Ki-67 exhibited immunopositivity ranging between 2 and 3% of cells in PA cases, 30% of neoplastic cells in PAC, and 70% in ACC neoplastic cells (Figure 2).

Immunostaining for p53 was approximately 30% in PA cases. High nuclear staining (60%) was observed in PAC cells, while 50% of the neoplastic cells displayed high nuclear immunoreactivity in ACC cases (Figure 2).

**Figure 3:**
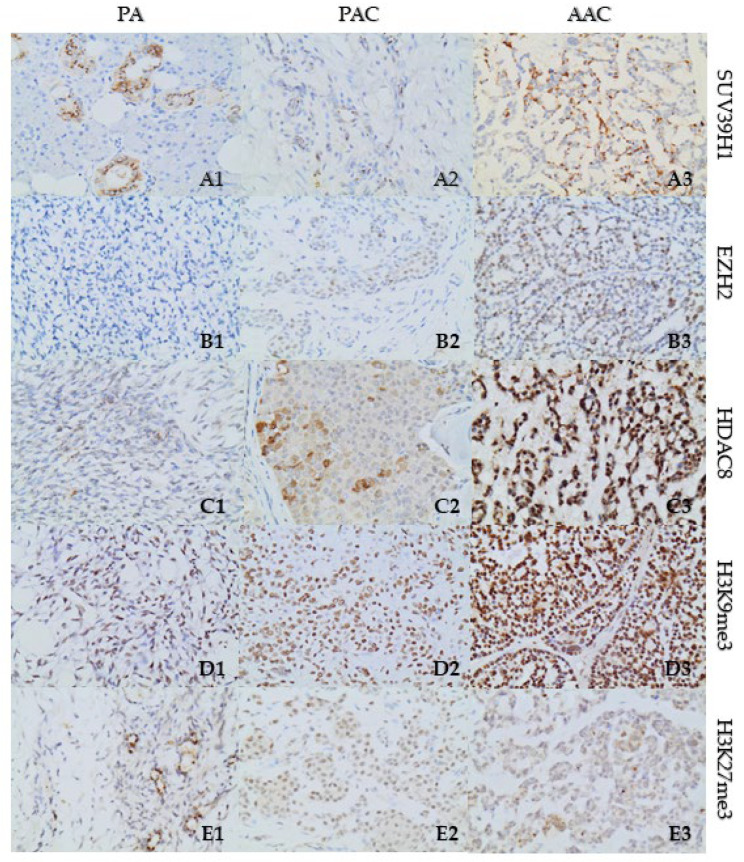
Representative immunostainings of SUV39H1, EZH2, HDAC8, H3K9me3, and H3K27me3 in a PA case (**A1**–**E1**), in a PAC case (**A2**–**E2**), and in an ACC case (**A3**–**E3**). Magnification ×400.

Immunostaining for EZH2 antibody was negative to weak (5%) in PA cases. Nuclear EZH2 staining was weak (15%) in PAC cases, and high (70%) in ACC cases (Figure 3).

Immunoreactivity for SUV39H1 was cytoplasmic in all tissues. In PA cases, SUV39H1 staining demonstrated negative-to-weak positivity in 20% of neoplastic cells, while in PAC tissues the positive staining, accounting for 2% of the neoplastic cells, was weak. In ACC tissues, SUV39H1 immunoreactivity was strong, manifesting cytoplasmic and paranuclear stain with punctate staining type, in 40% of the neoplastic cells (Figure 3).

Regarding HDAC8 immunostaining, PA cases exhibited nuclear and cytoplasmic staining in 10% of the neoplastic cells demonstrating high immunoreactivity, especially in the periphery of the tumor, while the staining of the internal control (ducts) was strong. In PAC cases, HDAC8 immunoreactivity was high, mainly cytoplasmic, and focally nuclear, in 40% of tumor cells. In ACC cases, HDAC8 immunoreactivity was positive in 50% of cells, displaying mainly high nuclear positivity and focally intense in the case of cytoplasmic positivity (Figure 3).

### 3.3. Increased Expression of Repressive Histone Marks H3K27me3 and H3K9me3 in SGT Tissues

Following the elevated expression of SUV39H1 and EZH2, we proceeded to investigate their activity regarding the deposition of repressive histone marks, H3K9me3 and H3K27me3 in SGT tissues, respectively.

Immunohistochemical staining of H3K9me3 showed weak positivity (40%) in the PA cases and high immunopositivity in 95% of the cells in PAC cases. In ACC tissues, high immunoreactivity was detected in 98% of the cells (Figure 3).

Furthermore, H3K27me3 immunoreactivity showed focal/weak positivity regarding PA cases (15%). Immunoreactivity in neoplastic cells of PAC cases was weak (80%), while the ACC cases demonstrated weak regional positivity (20%) (Figure 3). All immunostaining data are summarized in Table 3.

## 4. Discussion

Salivary gland tumors are rare neoplasms but are often aggressive, metastatic, and highly resistant to systemic therapy. In search of novel molecular targets to serve as predictive biomarkers, epigenetic factors have emerged as potential areas of research. The current study enriches previous scientific data pointing towards the implication of DNA and histone post-translational modifications in the pathogenesis of SGTs by performing a detailed bioinformatic analysis of a large cohort of ACC tumors and generating a PPI network of key epigenetic modifiers implicated in SGTs.

Notably, a significant upregulation of TP53 and the histone-modifying enzymes SUV39H1, EZH2, PRMT1, HDAC8, and KDM5B, along with the upregulation of DNA methyltransferase DNMT3A and ubiquitin ligase UHRF1 mRNA levels was detected in ACC tissues with downregulation of lysine acetyltransferase KAT2B levels. The protein expression of p53, SUV39H1, EZH2, and HDAC8 was further validated in SGT tissues along with the corresponding repressive histone marks H3K9me3 and H3K27me3.

A contributing role of p53 to the pathology of SGTs has been previously suggested, as it is often highly expressed in MECs and higher-grade ACCs [8,9,10,11,12]. Modifications of p53 have been associated with the transformation process to a higher grade, often due to mutations of TP53 or heterozygosity loss on gene loci and p53 protein overabundance on higher-grade ACCs than conventional morphology [8,9]. TP53 mutations are often detected in 5–10% of ACC cases, being mainly missense and resulting in loss of function [38,39,40,41]. TP53 mutations have been further associated with recurrence-free survival, overall survival, and metastatic propensity [41]. Moreover, perineural invasion which enhances epithelial–mesenchymal transition (EMT) is an essential event of ACCs and has been associated with p53 expression, suggesting a regulatory role in ACC metastatic phenomena [42]. In our study, nuclear p53 expression was low in PA cases and high in PACs and ACCs, confirming previous investigations.

With regard to histone-modifying enzymes, upregulation of the three key methyltransferases SUV39H1, EZH2, and PRMT1 was revealed in ACCs. SUV39H1 (suppressor of variegation 3–9 homolog 1) is a lysine methyltransferase which has been associated with several important events, including telomere maintenance, cell delineation, DNA-damage repair, and senescence. It is overexpressed in many cancer subtypes with relatively poor prognosis [43]. It is considered one of the most significant histone methyltransferases (HMTs) that mediates the heterochromatic H3K9me3 repressive mark, being involved in gene silencing [44]. In oral cancer, nuclear SUV39H1 expression has been correlated with tumor stage and progression [45].

The present study is the first to demonstrate increased mRNA and expression levels of SUV39H1 in ACCs compared to PA and PAC tissues. Following this observation, we also investigated the levels of the repressive mark H3K9me3 mediated by SUV39H1 in tissues and detected elevated expression in PAC and ACCs compared to PA, indicating a potential functional role of SUV39H1 in these tumors that is mediated through the increased H3K9me3 levels. In agreement, Lam-Ubol and colleagues have shown increased H3K9me3 expression in MECs and ACCs that was correlated with the proliferative and aggressive phenotype of tumors [24]. Moreover, upregulation of the H3K9me3 protein mark in primary ACCs was associated with poor prognosis and distant metastasis, serving as a potent prognostic biomarker. In addition, high H3K9me3 levels were associated with downregulation of H3K9ac expression in highly proliferative and aggressive ACCs. It has been proposed that H3K9 trimethylation and acetylation might modulate the expression of cell-proliferation-associated genes (*Cip1*/*p21*, *Notch1*, *ERK*), acting antagonistically in gene silencing, and being implicated in poor survival [46].

Histone methyltransferase EZH2 (enhancer of zeste homologue 2) is a core protein of the polycomb repressive complex-2 (PRC2) which catalyzes H3K27me3, leading to chromatin condensation and suppression of genes related to cell development and cell cycle and differentiation [47]. EZH2 has been shown to act in synergy with HDACs to regulate the suppression of gene transcription through H3K27me3 marks [48]. In salivary ACCs, upregulation of EZH2 has been associated with aggressive behavior and worse survival, presenting a crucial prognostic factor of recurrence [49]. High EZH2 expression (>25% immunoreactivity) has been corelated with significant deregulation of the cell cycle proteins p16INK4a, p53, E2F1, and cyclin D1, contributing to ACC onset [49]. Additionally, Chen and colleagues have demonstrated the significant role of EZH2-mediated H3K27me3 in ACC metastasis and poor prognosis by inactivating essential metastasis suppressors, such as E-cadherin [50]. In our study, EZH2 immunoreactivity was weak in PA cases and in PACs and increased in ACCs, while H3K27me3 expression was observed in all cases to be higher in PACs.

PRMT1 (protein arginine N-methyltransferase 1) is associated with DNA damage pathways and epigenetic modifications that repair DNA damage and ensure genomic stability [51]. PRMT1 is expressed in low levels in salivary glands [52] and its role in ACC’s pathogenesis has not been previously investigated. Our study is the first to indicate an upregulation of PRMT1 in ACCs and its functional role needs further elucidation. However, there is evidence that PRMT1 can act in synergy with CARM1 and p300 to drive p53 transcription [53]. Moreover, an association of PRMT1 overexpression with EZH2 has been involved in the establishment of malignant phenotype with poor outcomes [54].

Moving towards the deacetylation enzymes, only HDAC8 was found to be significantly upregulated in ACCs. HDAC8 deacetylation activity on histones H3 and H4 has been previously associated with the inhibition of the transcription of various genes, including *SIRT7*, *IFNB1*, *CCL4*, *SOCS-1/3*, *MAP2K3*, *ID2*, *MLN64*, and *BNIP3* [55]. Through its interaction with substrates that include histone or nonhistone proteins, HDAC8 exerts pluripotent effects enabling malignant development by regulating proliferation, apoptosis, epithelial/mesenchymal transitions (EMT) invasion, and immunity [55]. HDAC8 has been shown to deacetylate the tumor suppressor p53 at lysine residues (Lys381/382) [55] as well as interact with EZH2 and consequently suppress Wnt antagonists through histone H3K27 trimethylation and H4 deacetylation [56].

HDAC8 overexpression has been detected in oral squamous cell carcinoma (OSCC) tissues and its silencing was shown to significantly reduce cell proliferation and induce apoptotic cell death [57]. Inhibition of HDAC8 by the deacetylase inhibitor apicidin was further shown to induce cell growth inhibition and enable apoptosis as well as autophagy in murine OSCC and in MEC, indicating HDAC8 inhibition as a new agent in oral cancer [58]. The role of HDAC8 in salivary ACC has not been previously investigated and our study was the first to detect elevated mRNA and protein levels of HDAC8 in ACC and PAC cases compared to PAs, indicating the need for further investigation.

Concerning DNA methylation, increased mRNA levels of DNMT3A were detected in ACCs. DNMT3A is involved in the establishment of new methylation marks, inactivating the expression of new genes [59,60]. Previous studies have shown upregulation of DNMT3A in oral and lip lesions and association with malignant developments in the oral and maxillofacial area [61,62,63]. To date, information about the expression of DNMT3A in SGTs remains scarce. A single study has shown that in minor SGTs, DNMT3A shows mainly nucleic distribution and that nuclear immunohistochemical staining is observed in almost 17% of ACCs [64].

Another upregulated gene in the PPI network was UHRF1 (ubiquitin like with PHD and ring finger domains 1) which binds hemimethylated DNA and is involved in the fidelity of DNA methylation [65]. UHRF1 is linked to both methylation of DNA and acetylation/deacetylation of histones, therefore exerting the roles of chromatin reader and writer [65,66]. UHRF1 expression increases in malignancies but its role in oral cancer and salivary gland malignancies remains understudied. Investigation of the mutational landscape of ACCs revealed that besides *TP53*, the *UHRF1* gene was also involved in modifications in ~10% of the cases (homozygous deletions/mutations) [23]. In ACCs, dysregulation of casein kinase 1 (CK1) isoforms (-ε, -d), a member of the Wnt/b-catenin signaling pathway, has been detected [67], possibly affecting UHRF1′s stability [68] and leading to reduced DNA methylation and IFN-induced activation of p53 [69]. Moreover, upregulation of UHRF1 in malignancies has been suggested to involve interactions with DNA methyltransferases [70,71].

The lysine-specific demethylase-5B or histone demethylase-JARID1B encoded by the *KDM5B* gene expression was also increased in ACCs. KDM5B/PLU-1/JARID1B is a multidomain protein, involved in protein–protein interactions as well as gene and chromatin modifications. It is a potent transcriptional suppressor which interacts with the chromatin and has a limited expression in physiological human tissues [72]. KDM5B is a co-activator rather than a co-suppressor of retinoic acid (RA), and their interaction is essential for RA-related gene expression. In the nucleus, KDM5B associates directly with EZH2 and regulates the RA signaling pathway [73]. An association of JARID1 members with HDACs has also been reported to regulate their induction [72].

KDM5B is required for stem cell function in oral cancer, and KDM5B-high cells are a definite sub-population in oral carcinoma that exhibits typical cancer stem cell (CSC)-related markers and PI3K signaling activation [74]. By validating the effect of H3K4 methylation in salivary gland mucinous adenocarcinoma (SMAdC), it was revealed that ~40% of the upregulated proteins were related to the KDM5B effect. Overall, 21% of KDM5B-associated genes were related to cell cycle regulation, and others to mitosis, DNA repair, and chromatin remodeling. Additionally, 10% of KDM5B-associated genes were associated with the regulation of cancer stem cells and the rest (17%) were related to malignant behavior and poor tumor prognosis [16]. Nonetheless, to our knowledge, the impact of KDM5B on ACCs has not yet been evaluated.

Regarding downregulated genes, the bioinformatic analysis revealed reduced mRNA levels of the lysine acetyltransferase KAT2B/PCAF which belongs to the GNAT (GCN5-associated N-acetyltransferase) family. KAT2B binds CBP and p300 to exert histone acetylation and gene transcription [75,76]. KAT2B regulates cell cycle growth, gluconeogenesis, cell differentiation, angiogenesis, and tumorigenesis [77,78]. There is evidence that it might inhibit vascular senescence [79] and malignant transformation through PTEN regulation [80], TP53, or acetylation [81,82]. KAT2B is directly associated with the p53/p21 pathway, and it can regulate the p53-related expression of p21, but not p300 or CBP, due to various p53-triggered stresses, through histone H3 acetylation [83]. KAT2B-related acetylation of EZH2 blocks its ability for target gene regulation, resulting in cancer progression, and is associated with poor survival [84]. In salivary adenocarcinoma, CBP functions as a transcriptional co-activator of RA which provokes cell proliferation and differentiation [85], suggesting a similar potential role of KAT2B. However, KAT2B has not been previously investigated in SGTs and the significant downregulation observed in this study indicates a potential gene regulation mechanism that needs further investigation.

## 5. Conclusions

In conclusion, our study revealed a network of interacting histone-modifying factors implicated in the gene regulation of SGTs, highlighting the crucial role of epigenetic mechanisms in salivary gland tumorigenesis. Among the differentially expressed genes identified in ACCs, the pivotal methyltransferases SUV39H1 and EZH2, as well as the deacetylase HDAC8, were further detected to be overexpressed in tissue samples, validating the PPI network as well as some previous studies [49,50]. Moreover, increased expression of the repressive histone marks H3K9me3 and H3K27me3 in tissues indicates a potential functional role of SUV39H1 and EZH2, respectively, with a negative impact in gene regulation.

Further studies are required to confirm the potential physical interactions among the detected histone-modifying factors, as well as to elucidate the molecular mechanisms and the functional role of the identified differentially expressed genes in salivary gland tumors.

## Figures and Tables

**Table 1:** Characteristics of antibodies used in immunohistochemical analysis.

Primary Antibody	Company	Catalogue No.	Clone	Host	IHC Dilution	Antigen Retrieval	Positive Control
Ki-67	Dako	M7240	Monoclonal	Mouse	1:200	pH = 9	-
p53	Dako	M7001	Monoclonal	Mouse	1:200	pH = 6	-
SUV39H1	EMD Millipore	05-615	Monoclonal	Mouse	1:300	pH = 6	Human breast cancer
Ezh2	Abcam	Ab283270	Monoclonal	Mouse	1:100	pH = 9	Human breast cancer
HDAC8	Abcam	Ab217702	Polyclonal	Rabbit	1:150	pH = 6	Human brain
H3K27me3	Millipore, MA	07-449	Polyclonal	Rabbit	1:50	pH = 6	Human normal breast
H3K9me3	BioLegend	815601	Monoclonal	Mouse	1:80	pH = 6	Human breast

**Table 2:** The table depicts the genes *EZH2*, *PRMT1*, *KDM5B*, *TP53*, *HDAC8*, *SUV39H1*, *UHRF1*, *KAT2B*, and *DNMT3A* participating in the PPI network. Included in the table are the Ensembl IDs, corresponding gene symbols, adjusted *p*-values, and Log_2_FCs.

Ensembl ID	Gene Symbol	Adj. *p*-Value	Log_2_FC
ENSG00000276043	UHRF1	1.72 × 10^−2^	2.638120288
ENSG00000101945	SUV39H1	3.52 × 10^−2^	2.243544044
ENSG00000141510	TP53	1.78 × 10^−2^	2.1709429
ENSG00000106462	EZH2	5.29 × 10^−3^	1.8144852
ENSG00000126457	PRMT1	9.91 × 10^−3^	1.58807423
ENSG00000119772	DNMT3A	1.85 × 10^−2^	1.524719521
ENSG00000117139	KDM5B	6.76 × 10^−3^	1.400579007
ENSG00000147099	HDAC8	1.24 × 10^−2^	1.297709246
ENSG00000114166	KAT2B	2.49 × 10^−2^	−2.244361094

**Table 3:** Immunohistochemical expression of Ki-67, p53, H3K27 trimethylation, EZH2, SUV39H1 and HDAC8 in SGTs.

Antibody	SGT Immunoreactivity		
	PA	PAC	ACC
Ki-67	2–3%	30%	70%
p53	30%; weak nuclear	60%; high nuclear	50%; high nuclear
EZH2	5%; weak nuclear	15%; weak nuclear	70%; high nuclear
SUV39H1	20%; weak cytoplasmic	2%; weak cytoplasmic	40%; high cytoplasmic and paranuclear stain with punctate staining type
HDAC8	10%; high nuclear and cytoplasmic	40%; high nuclear and cytoplasmic	50%; high nuclear and cytoplasmic
H3K9me3	40%; weak	95%; high	98%; high
H3K27me3	15%; focal, weak	80%; weak	20%; regional, weak

## Data Availability

The STRING analysis can be reached via the permanent link: https://version-11-5.string-db.org/cgi/network?networkId=bJlnNgyoEmk4 (accessed on 7 October 2023).

## Supplementary Materials

The following supporting information can be downloaded at: https://www.mdpi.com/article/10.3390/cells12202437/s1, Table S1: DEG list; Table S2: PPI network enriched terms.

## Abbreviations

ACC: adenoid cystic carcinoma; CK1: casein kinase 1; CSC: cancer stem cell; DAB: diaminobenzidine; DEGs: differentially expressed genes; DNMT3A: DNA methyltransferase 3 alpha; EMT: epithelial–mesenchymal transition; EZH2: enhancer of zeste homologue 2; FDR: false discovery rate; FFPE: formalin-fixed, paraffin-embedded; GNAT: GCN5-associated N-acetyltransferase; GO: gene ontology; GSEA: gene set enrichment analysis; HMTs: histone methyltransferases; HN: head and neck; Log2FC: log2 fold change; MEC: mucoepidermoid carcinoma; NGS: normal goat serum; NIH: National Institute of Health; ORA: over-representation analysis; OSCC: oral squamous cell carcinoma; PA: pleiomorphic adenoma; PAC: polymorphous adenocarcinoma; PPI: protein–protein interactions; PRC2: polycomb repressive complex-2; PRMT1: protein arginine methyltransferase 1; RA: retinoic acid; SG: salivary gland; SGTs: salivary gland tumors; SMAdC: salivary gland mucinous adenocarcinoma; SRA: sequence read archive; SUV39H1: suppressor of variegation 3–9 homolog 1; UHRF1: ubiquitin like with PHD and ring finger domains 1; WebGestalt: WEB-based gene set analysis toolkit; WHO: World Health Organization.
